# Impact of Smoking and Brain Metastasis on Outcomes of Advanced *EGFR* Mutation Lung Adenocarcinoma Patients Treated with First Line Epidermal Growth Factor Receptor Tyrosine Kinase Inhibitors

**DOI:** 10.1371/journal.pone.0123587

**Published:** 2015-05-08

**Authors:** Amit Jain, Cindy Lim, Eugene MingJin Gan, David Zhihao Ng, Quan Sing Ng, Mei Kim Ang, Angela Takano, Kian Sing Chan, Wu Meng Tan, Ravindran Kanesvaran, Chee Keong Toh, Chian Min Loo, Anne Ann Ling Hsu, Anantham Devanand, Chong Hee Lim, Heng Nung Koong, Tina Koh, Kam Weng Fong, Swee Peng Yap, Su Woon Kim, Balram Chowbay, Lynette Oon, Kiat Hon Lim, Wan Teck Lim, Eng Huat Tan, Daniel Shao Weng Tan

**Affiliations:** 1 Division of Medical Oncology, National Cancer Centre Singapore, Singapore, Singapore; 2 Clinical Trials & Epidemiological Sciences, National Cancer Centre Singapore, Singapore, Singapore; 3 Yong Loo Lin School of Medicine, National University of Singapore, Singapore, Singapore; 4 Department of Pathology, Academia, Level 7, Singapore General Hospital, Singapore, Singapore; 5 Respiratory and Critical Care Medicine, Singapore General Hospital, Singapore, Singapore; 6 Department of Cardiothoracic Surgery, National Heart Centre Singapore, Singapore, Singapore; 7 Division of Surgical Oncology, National Cancer Centre Singapore, Singapore, Singapore; 8 Division of Radiation Oncology, National Cancer Centre Singapore, Singapore, Singapore; 9 Clinical Pharmacology Laboratory, Division of Medical Sciences, National Cancer Centre Singapore, Singapore, Singapore; 10 Cancer Therapeutics Research Laboratory, Division of Medical Sciences, National Cancer Centre Singapore, Singapore, Singapore; 11 Cancer Stem Cell Biology, Genome Institute of Singapore, Singapore, Singapore; Queen Mary Hospital, HONG KONG

## Abstract

**Objectives:**

This purpose of this study was to examine clinical-pathologic factors – particularly smoking and brain metastases – in *EGFR* mutation positive (M+) lung adenocarcinoma (ADC) to determine their impact on survival in patients treated with first line EGFR TKI.

**Methods:**

A retrospective review of *EGFR* mutation reflex testing experience for all ADC diagnosed at a tertiary Asian cancer centre from January 2009 to April 2013. Amongst this cohort, patients with advanced *EGFR* M+ ADC treated with first line EGFR TKI were identified to determine factors that influence progression free and overall survival.

**Results:**

444/742 (59.8%) ADC reflex tested for *EGFR* mutations were *EGFR* M+. Amongst never-smokers (n=468), *EGFR* M+ were found in 74.5% of females and 76.3% of males, and amongst ever smokers (n=283), in 53.3% of females and 35.6% of males. Exon 20 mutations were found more commonly amongst heavy smokers (> 50 pack years and > 20 pack years, Pearson’s chi square p=0.044, and p=0.038 respectively). 211 patients treated with palliative first line TKI had a median PFS and OS of 9.2 and 19.6 months respectively. 26% of patients had brain metastasis at diagnosis. This was significantly detrimental to overall survival (HR 1.85, CI 1.09-3.16, p=0.024) on multivariate analysis. There was no evidence that smoking status had a significant impact on survival.

**Conclusions:**

The high prevalence of *EGFR* M+ in our patient population warrants reflex testing regardless of gender and smoking status. Smoking status and dosage did not impact progression free or overall survival in patients treated with first line EGFR TKI. The presence of brain metastasis at diagnosis negatively impacts overall survival.

## Introduction

EGFR tyrosine kinase inhibitors (TKI) such as gefitinib and erlotinib, are now established first line treatment options for *EGFR* mutation positive (*EGFR* M+) lung adenocarcinoma (ADC), demonstrating significant improvement in progression free survival (PFS) over platinum-based doublet chemotherapy [1–7]. Previous studies examining the impact of smoking history on TKI response often reflect surrogacy for *EGFR* mutations and majority of phase III studies were enriched for never smokers. A recent retrospective study suggested that smoking history and smoking dosage may be associated with significantly poorer response rates and survival outcomes in EGFR mutation positive non-small cell lung cancer (NSCLC) [8]. However, this finding is confounded by the fact that a greater proportion of smokers had received EGFR TKI beyond the second and third line setting, and the impact of smoking on survival in *EGFR* mutation positive NSCLC patients receiving first line EGFR TKI remains unclear [9].

Due to the high incidence of *EGFR* mutations in Asian ADC compared to the West [10–11], many academic hospitals, including our centre, have adopted reflex testing for *EGFR* mutations. As cost effectiveness of EGFR TKI is driven by patient selection based on *EGFR* mutation status [12], it is important to define the prevalence of the mutation in both smokers (current and ex-smokers) as well as never smokers through systematic testing of consecutive cases. Clinical pathologic factors such as smoking status [8], location of *EGFR* mutation [13], and presence of brain metastases [14] may impact on treatment outcomes. Of particular interest, brain metastasis in *EGFR* mutation positive NSCLC is a common site of involvement at diagnosis and treatment failure—occurring in up to 23% of newly diagnosed patients [15]. Elucidating prognostic factors in *EGFR* mutant ADC treated with first line TKI will facilitate improved stratification and identify therapeutically challenging patient subgroups.

In this study, we report our reflex *EGFR* testing experience on consecutive lung adenocarcinomas seen in an Asian tertiary cancer centre and determine the prevalence of *EGFR* mutations by gender and smoking status. Relationships between mutation spectra and clinical characteristics such as age, gender, ethnicity and smoking status were also explored. Further, in those who had received first line treatment with an EGFR TKI, we examined clinical pathologic characteristics that had an impact on survival.

## Materials and Methods

### Study Population

Prior to 1st June 2010, *EGFR* mutation testing in our centre for patients with newly diagnosed ADC was ordered as per physician discretion. From 1st June 2010 all ADC samples identified by the pathologists were reflex tested for *EGFR* mutations, regardless of stage and smoking status.

Smoking status for patients was obtained from electronic medical records and Lung Cancer Consortium Singapore, where patients’ lifestyle factors were captured through interviews by research coordinators. Patients were classified as never smokers (NS), and ever smokers (ex-smokers [quit ≥ 1 year] and current smokers) (ES). NS were defined as those who had smoked less than 100 sticks in their lifetime.

Patients with Stage 4 *EGFR* M+ disease who had received TKI therapy were evaluated. Patients who had already been commenced on TKI based on their phenotypic traits (Asian, non-smoker), but subsequently underwent confirmatory *EGFR* mutation testing were also included. Baseline imaging was evaluated for presence or absence of brain metastases, and response evaluation scans were performed according to physician discretion, ranging from within 6 weeks from start of TKI for the first scan, to 8–12 weekly for subsequent scans. First line TKI treated *EGFR* M+ ADC patients were analysed for progression free survival (PFS) and overall survival (OS), and an exploratory analysis was done for factors that influenced survival.

### EGFR Mutational Analysis

*EGFR* mutation analysis was carried out by Sanger sequencing on genomic DNA that had been extracted from formalin fixed paraffin embedded (FFPE) tissue samples using Qiagen FFPE DNA extraction kit. Extracted DNA was then subject to polymerase chain reaction (PCR) amplification of exons 18, 19, 20 and 21, and products were analysed by direct Sanger sequencing.

### Statistical Analysis

Continuous clinical variables were summarized using median and range, while categorical variables were summarized by frequency and percentage. Pearson’s chi-squared test (or Fisher’s exact test if there were expected cell frequencies less than 5) was used to test for associations between patient characteristics and mutation type, smoking status and presence of brain metastasis at diagnosis. The strength of the association was estimated using Cramer’s V.

PFS was calculated as the duration from start of TKI to the PFS date defined by clinico-radiological progression of disease on CT imaging as per RECIST 1.1. OS was calculated as the duration of time from start of TKI to date of demise. The Kaplan-Meier method was used to estimate survival functions. The log-rank test was used to determine if there was a difference in survival curves between different groups. A 2-sided p-value of less than 0.05 was taken as statistically significant. Univariate and multivariate analysis was performed using the Cox proportional hazards model. All analyses were performed in Stata (Version 12.1; StataCorp, Texas, USA).

### Ethics

This research was approved by SingHealth CIRB (CIRB 2010/516/B) and all clinical investigation was conducted according to the principles expressed in the Declaration of Helsinki. The data was collected and analysed anonymously then reported.

## Results

### Reflex Testing Experience

A total of 994 cases of ADC were seen in our centre between 1st January 2009 and 18th April 2013. Reflex testing was adopted from 1^st^ June 2010. A 6 monthly review from 1^st^ June 2010 to 19^th^ April 2013 revealed that 87–94% of all ADC cases were tested, with an *EGFR* M+ rate of 50–68% (S1 Table). A total of 742 patients were tested for *EGFR* mutations. 444 (59.8%) were positive and 289 (38.9%) negative for mutations. 9 cases were unsuccessfully profiled due to incomplete sequencing (n = 5: unsuccessful for Exon 18, n = 1; Exon 20, n = 2; Exons 20/21, n = 1 and Exons 19/20/21, n = 1), tumor content of less than 15% raising the possibility of a false negative result (n = 1) or insufficient tissue for any analysis (n = 4). None of these patients underwent a repeat biopsy and they were treated as for ADC that was *EGFR* mutant negative. Hence the ascertainment rate with *EGFR* sequencing in our centre was 98.8%.

### Smoking Status, Sex and *EGFR* Mutation Status of 762 ADC

Amongst the 762 cases evaluated for *EGFR* mutations, there were 464 NS (334, 72.0% females; 130, 28.0% males), 282 ES (30, 10.6% females; 252, 89.4% males) and 11 patients with unknown smoking status (6 females and 5 males).

**Fig 1:**
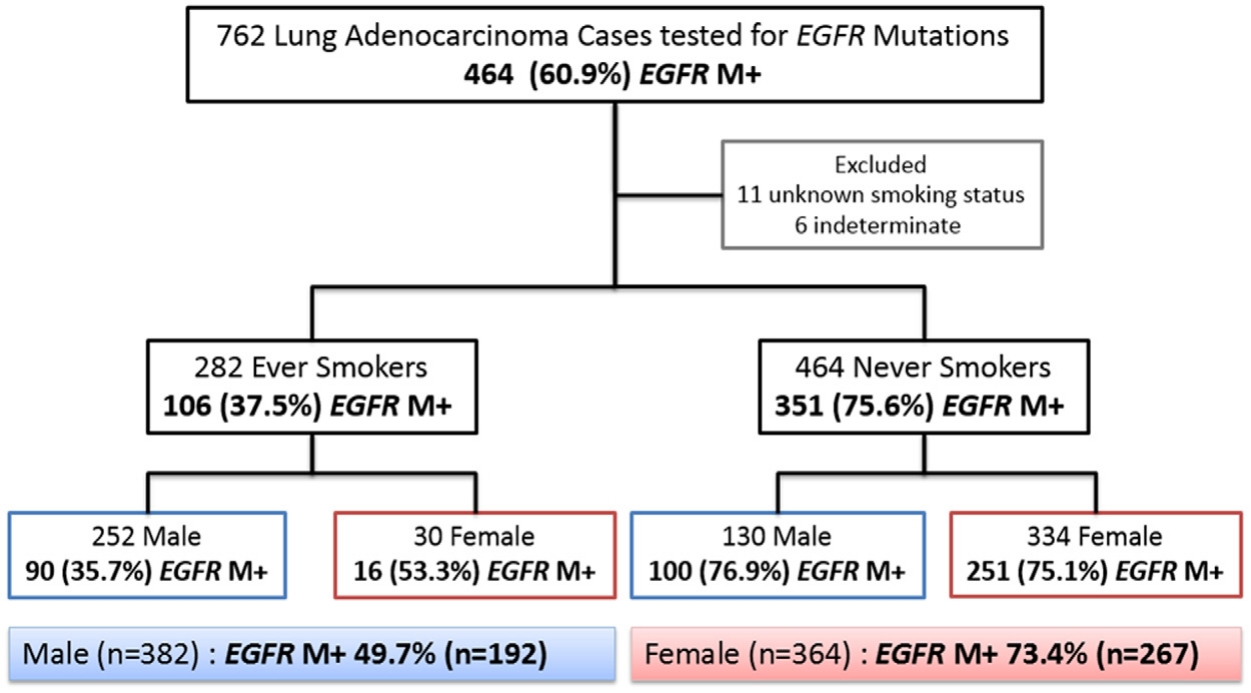
Clinical characteristics and *EGFR* mutation status rates categorised by smoking status and sex. 11 patients with unknown smoking status, and 6 who had samples indeterminate for *EGFR* mutational status were excluded. 464/762 (60.9%) tested positive for *EGFR* mutations (*EGFR* M+). The number of patients needed to test in order to pick up 1 *EGFR* mutant lung adenocarcinoma in any sub-population stratified by sex and smoking status, was less than 3 patients (male ES; 1/0.357 = 2.8).

**Fig 2:**
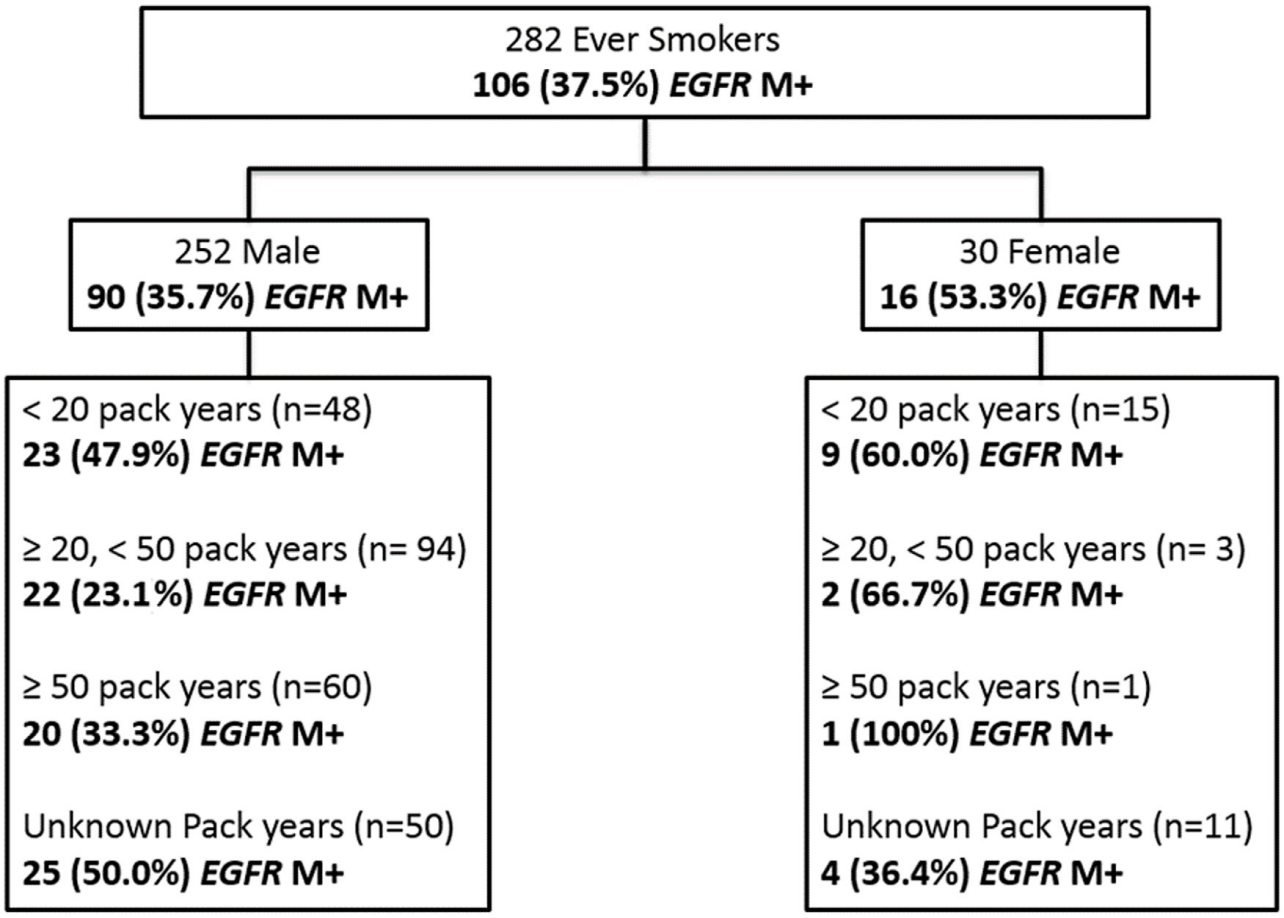
*EGFR* mutation rates amongst ever smokers classified by pack years.

The respective *EGFR* M+ rates amongst female NS, female ES, male NS and male ES were 75.1%, 53.3%, 76.9%, and 35.7% (Fig 1). ES were further stratified by pack years based on available information regarding their smoking histories. *EGFR* M+ rates were at least 20 percent in both male and female heavy smokers but further interpretation was limited by the small numbers in these sub-groups (Fig 2).

### EGFR mutations, Age, Sex, Ethnicity, Smoking Status

A total of 464 *EGFR* M+ patients were seen at our centre. 444 of the 464 *EGFR* M+ patients were tested at our centre and 17 were tested in another College of American Pathologist (CAP) accredited centres with details of mutational testing available. The remaining 3 were documented in clinical notes to have tested positive for exon 19 deletions in other external laboratory but further details of analysis were not available.

**Fig 3:**
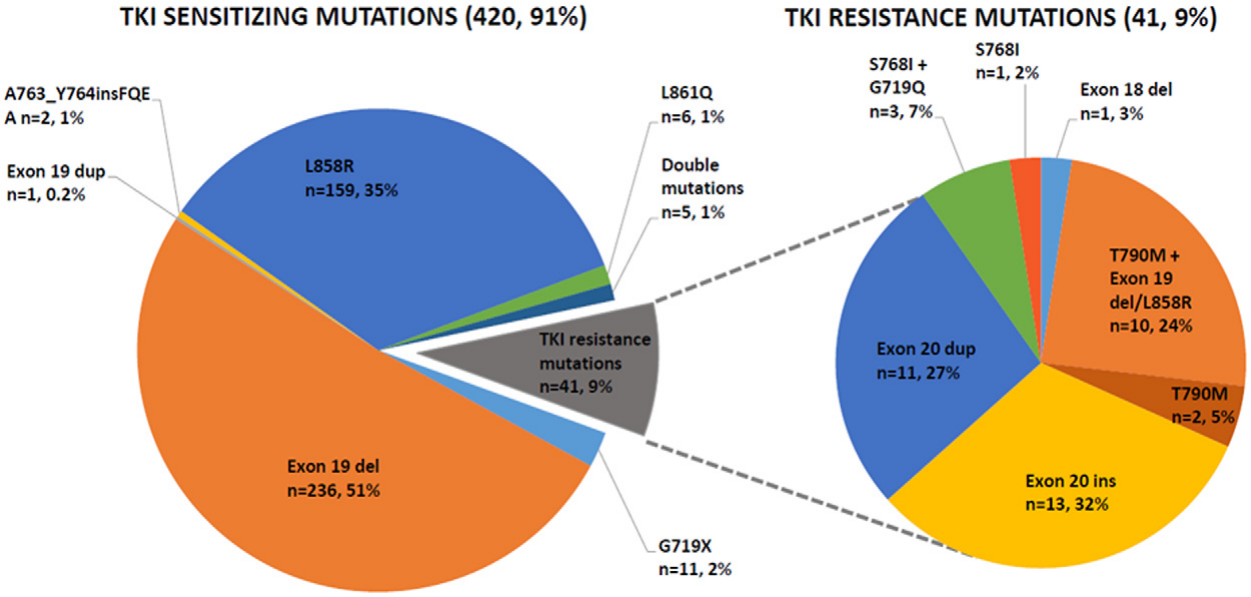
Sites of EGFR mutations amongst 461 patients.

Of 461 *EGFR* M+, 414 (89.8%) harboured TKI sensitising mutations and 47 (10.1%) harboured mutations known to confer resistance to *EGFR* TKI. Single mutations in *EGFR* were detected in exon 18 (n = 12, 2.6%), exon 19 (n = 239, 51.8%), exon 20 (n = 29, 6.2%), and exon 21 (n = 166, 36.0%) Exon 18 mutations comprised 11 G719X mutations (8 G719A, 2 G719S, and 1 G719C), and 1 Exon 18 del (p.E709_T710>D). Exon 19 mutations comprised deletions of between 10 to 24 base pairs with or without 1) additional insertion of between 1 to 3 base pairs, or 2) substitution of 1 to 2 base pairs. The most prevalent mutations were deletions c.2235_2249del15 (n = 95, 39.7% of all Exon 19 mutations), followed by c2236_2250del15 (n = 41, 17.1% of all Exon 19 mutations). Exon 20 mutations comprised 13 insertion (51.7% of Exon 20 mutations), 11 duplications (37.9% of Exon 20 mutations) 2 T790M point mutations, 1 S768I point mutation, and 2 sensitising A763_Y764insFQEA insertion mutations. Exon 21 mutations comprised L858R (n = 159, 95.8% of all Exon 21 mutations), and L861Q (n = 6, 3.6% of all Exon 21 mutations) (Fig 3).

15 (3.2%) had dual *EGFR* mutations: 10 with Exon 20 mutations (5 T790M & Exon 19 del; 2 T790M & L858R; 3 S768I & G719Q), 3 with G719Q & L858R, 1 Exon 19 del & L833V (Exon 21 mutation), and 1 Exon 19 del & L858R.

Exon 19 deletions were seen in younger (<65 years) patients while exon 21 mutations were relatively more common in old (≥65 years) patients (WHO criteria) (59.1% vs. 42.3%, p = 0.002, Cramer’s V = 0.181; weak association). There was no evidence of any association between sex and type of mutation (p = 0.756) or exon(s) involved (p = 0.136), or between ethnicity (Chinese vs. others) and type of mutation (p = 0.784) or exon(s) involved (p = 0.579). There was a trend towards never smokers harbouring mutations in exon 19, while more ever-smokers had mutations in exon 20 but this association was not significant (p = 0.119).

### Impact of smoking dosage and *EGFR* mutations

Heavy smokers with more than 50 pack years appeared to have more mutations in exon 20 than those with less pack years or who had never smoked (p = 0.044, Cramer’s V = 0.145; weak association). A less stringent definition for heavy smokers (>20 pack years) still showed a significant association (18% vs. 6%, p = 0.038, Cramer’s V = 0.155; weak association). There was no significant association between type of mutation (indel vs. point) and heavy smoking status.

### Use of EGFR TKI amongst *EGFR* M+ ADC

461 patients with *EGFR* M+ ADC comprised 57 (12.3%) Stage I, 20 (4.3%) Stage II, 49 (10.7%) Stage IIIA/B and 335 (72.7%) Stage IV disease. 121 were treated with curative intent. 294 patients received palliative systemic therapy of which 211 (71.8%) received first line TKI, 55 (18.7%) received 1^st^ line chemotherapy, 28 (9.5%) received a combination of TKI and other agents including chemotherapy and combination targeted therapy in a phase I setting. 46 patients were censored as they were either lost to follow up, on best supportive care, declined treatment or passed away prior to commencing TKI. Of the 55 patients who did not receive TKI in the first line, 33 went on to receive TKI as second line therapy, 5 as third line, and 2 as fourth line. The remaining 15 never received *EGFR* TKIs in their lifetime. In total, 279 out of 294 (94.9%) *EGFR* M+ ADC received systemic therapy with a TKI in their lifetime.

### Clinical characteristics of EGFR TKI treated Stage IV NSCLC

A homogenous population of 211 patients with Stage IV disease treated with first line TKI were evaluated for survival on TKI and smoking history and other clinical characteristics were analysed for impact on survival. The median age of the cohort was 62 (33–84). 128 (60.7%) were females. 166 (78.7%) had never smoked, 32(15.1%) were ex-smokers and 13 (6.2%) were smokers. Amongst ever smokers with accurate smoking histories (n = 32), the median number of pack years was 30 pack years (ranging from 0.9 to 102). 26% had proven brain metastasis at time of diagnosis. Other key characteristics are summarised in Table 1.

**Table 1:** Demographics of 211 patients treated with 1^st^ line TKI.

Variable	Number	%
Total number of patients	211	100
Age at diagnosis, years		
Median (range)	62 (33–84)	
≤ 65	128	60.7
> 65	83	39.3
Sex		
Female	128	60.7
Male	83	39.3
Ethnicity		
Chinese	179	84.8
Malay	19	9.0
Indian	4	1.9
Others	9	4.3
Smoking status		
Never	166	78.7
Ex	32	15.2
Current	13	6.2
Smoking pack-years (amongst ever-smokers only)		
Median (range)	30 (0.9–102)	
Unknown	13	28.9
Smoking pack-years (including never smokers)		
< 50	188	89.1
≥ 50	10	4.7
Unknown	13	6.2
Smoking pack-years (including never smokers)		
< 20	178	84.4
≥ 20	20	9.5
Unknown	13	6.2
ECOG at diagnosis		
0	78	37.0
1	116	55.0
2	11	5.2
3	5	2.4
4	1	0.5
Brain metastasis at diagnosis		
No	156	73.9
Yes	55	26.1
Type of mutation		
Exon 21 L858R mutation	72	34.1
Exon 19 deletions	114	54.0
Exon 18 mutations (G719X)	2	1.0
Exon 21 L861Q	4	1.9
T790M mutation	5	2.4
Exon 20 mutations (other than T790M)	7	3.3
Double mutations (other than those containing T790M)	2	1.0
Others	1	0.5
Unknown	4	1.9
First line TKI		
Gefitinib	192	91.0
Erlotinib	10	4.7
Afatinib	9	4.3
Follow-up duration, months		
Median (range)	10.2 (0–78.0)	

Baseline characteristics were compared between patients who were never smokers vs. ever smokers and those without brain metastasis at diagnosis vs. those with brain metastasis at diagnosis (S2 and S3 Tables). There was a strong correlation between sex and smoking status (p<0.001, Cramer’s V = 0.576). 93.3% of ever smokers were male. Significant but weak associations were also observed between age and smoking status (p = 0.012, Cramer’s V = 0.173), type of mutation and smoking status (p = 0.044, Cramer’s V = 0.173), age and presence of brain metastasis (p = 0.014, Cramer’s V = 0.169), and ECOG performance status and presence of brain metastasis (p = 0.017, Cramer’s V = 0.181).

The progression free and overall survival of this specific population follows (Tables 2 and 3). Since the ever smokers were mostly male, smoking status was combined with sex into a single variable for multivariate analysis. Males were sub-categorised into never smokers and ever smokers.

**Table 2:** Univariate analysis of progression free survival and overall survival.

Variable	No. of events / No. of patients	Median PFS, months (95% CI)	P-value	No. of events / No. of patients	Median OS, months (95% CI)	P-value
	*Progression Free Survival*			*Overall Survival*		
All patients	114 / 210	9.2 (8.1–11.2)		74 / 210	19.6 (16.4–23.3)	
Age at diagnosis, years						
≤ 65	68 / 128	9.1 (6.7–10.8)		45 / 128	19.6 (16.0–26.0)	
> 65	46 / 82	10.6 (8.2–13.3)	0.390	29 / 82	19.0 (14.4–UND)	0.840
Sex						
Female	72 / 128	9.4 (7.6–11.4)		43 / 128	22.0 (17.6–UND)	
Male	42 / 82	9.2 (5.4–14.2)	0.522	31 / 82	16.0 (13.7–22.1)	0.237
Ethnicity						
Chinese	103 / 178	8.9 (7.2–10.6)		66 / 178	19.6 (16.0–23.3)	
Others	11 / 32	11.7 (7.4–UND)	0.093	8 / 32	28.8 (11.1–UND)	0.682
Smoking status						
Never	95 / 165	9.2 (7.6–11.2)		59 / 165	21.1 (17.1–26.0)	
Ever	19 / 45	11.4 (4.6–16.5)	0.844	15 / 45	16.0 (10.2–UND)	0.452
Smoking status						
Never	95 / 165	9.2 (7.6–11.2)		59 / 165	21.1 (17.1–26.0)	
Ex	15 / 32	10.6 (3.7–UND)		10 / 32	22.1 (10.2–UND)	
Current	4 / 13	16.5 (1.4–UND)	0.792	5 / 13	16.0 (2.8–UND)	0.380
Smoking pack-years						
< 50	102 / 187	9.4 (8.2–11.2)		64 / 187	21.1 (17.1–26.0)	
≥ 50	6 / 10	4.6 (0.4–UND)	0.510	4 / 10	16.0 (6.0–UND)	0.916
Smoking pack-years						
< 20	98 / 177	9.2 (8.1–11.4)		61 / 177	21.1 (17.1–26.0)	
≥ 20	10 / 20	10.6 (3.4–UND)	0.895	7 / 20	16.5 (9.3–UND)	0.906
ECOG at diagnosis						
0–1	101 / 193	9.7 (8.2–13.1)		63 / 193	22.0 (17.6–26.0)	
2–4	13 / 17	5.8 (3.8–10.6)	0.007	11 / 17	14.4 (5.8–15.8)	< 0.001
Brain mets at diagnosis						
No	81 / 155	10.4 (8.2–12.8)		47 / 155	22.1 (16.4–UND)	
Yes	33 / 55	8.2 (5.7–9.7)	0.053	27 / 55	17.9 (10.1–23.3)	0.029
Location of mutation						
Exon 19	58 / 115	9.7 (8.2–14.5)		43 / 115	17.9 (16.0–22.1)	
Exon 21	45 / 77	8.3 (5.9–11.6)		24 / 77	28.8 (14.4–UND)	
Others	10 / 16	2.1 (1.4–11.2)	0.013	7 / 16	22.0 (5.5–UND)	0.583
Type of mutation						
Exon 19 deletion	58 / 114	9.7 (8.2–14.5)		43 / 114	17.9 (16.0–22.1)	
Exon 21 L858R mutation	40 / 71	9.2 (7.0–11.7)		20 / 71	28.8 (14.7–UND)	
Others	13 / 21	4.6 (1.7–10.6)	0.019	10 / 21	15.6 (8.1–UND)	0.381

**Table 3:** Multivariate analysis of progression free survival and overall survival.

Variable	No. of events/ No. of patients	Hazard ratio (95% CI)	P-value	No. of events / No. of patients	Hazard ratio (95% CI)	P-value
	*Progression Free Survival*			*Overall Survival*		
Overall	111 / 206		0.009	73 / 206		0.032
Age						
≤ 65		1			1	
> 65		0.75 (0.49–1.14)	0.173		0.98 (0.59–1.64)	0.936
Gender and smoking status						
Female		1			1	
Male—never smoked		1.16 (0.72–1.86)	0.552		1.25 (0.68–2.28)	0.476
Male—ever smoked		0.74 (0.41–1.34)	0.327		1.11 (0.58–2.14)	0.756
Type of mutation						
Exon 19 deletion		1			1	
Exon 21 L858R mutation		1.27 (0.84–1.92)	0.258		0.69 (0.40–1.19)	0.186
Others		2.94 (1.44–5.99)	0.003		1.03 (0.48–2.22)	0.940
Brain metastasis at diagnosis						
No		1			1	
Yes		1.56 (0.99–2.45)	0.053		1.82 (1.07–3.11)	0.028
ECOG at diagnosis						
0–1		1			1	
2–4		2.77 (1.31–5.87)	0.008		4.40 (1.87–10.35)	0.001
Brain metastasis and ECOG interaction		0.49 (0.14–1.70)	0.264		0.36 (0.09–1.44)	0.147

### Progression Free Survival of EGFR TKI treated Stage IV Disease

The median PFS of all patients was 9.2 months (95% CI 8.1–11.2 months). Patients with single non-exon 19/21 mutations and double mutations had inferior PFS (p = 0.013) as compared to those with exon 19/21 mutations.

There was no significant difference in the PFS of ever (current and ex-), and never smokers (p = 0.844) nor between heavy smokers and others (p = 0.510 for ≥ 50 pack years, and p = 0.895 for ≥ 20 pack years) (Table 2).

**Fig 4:**
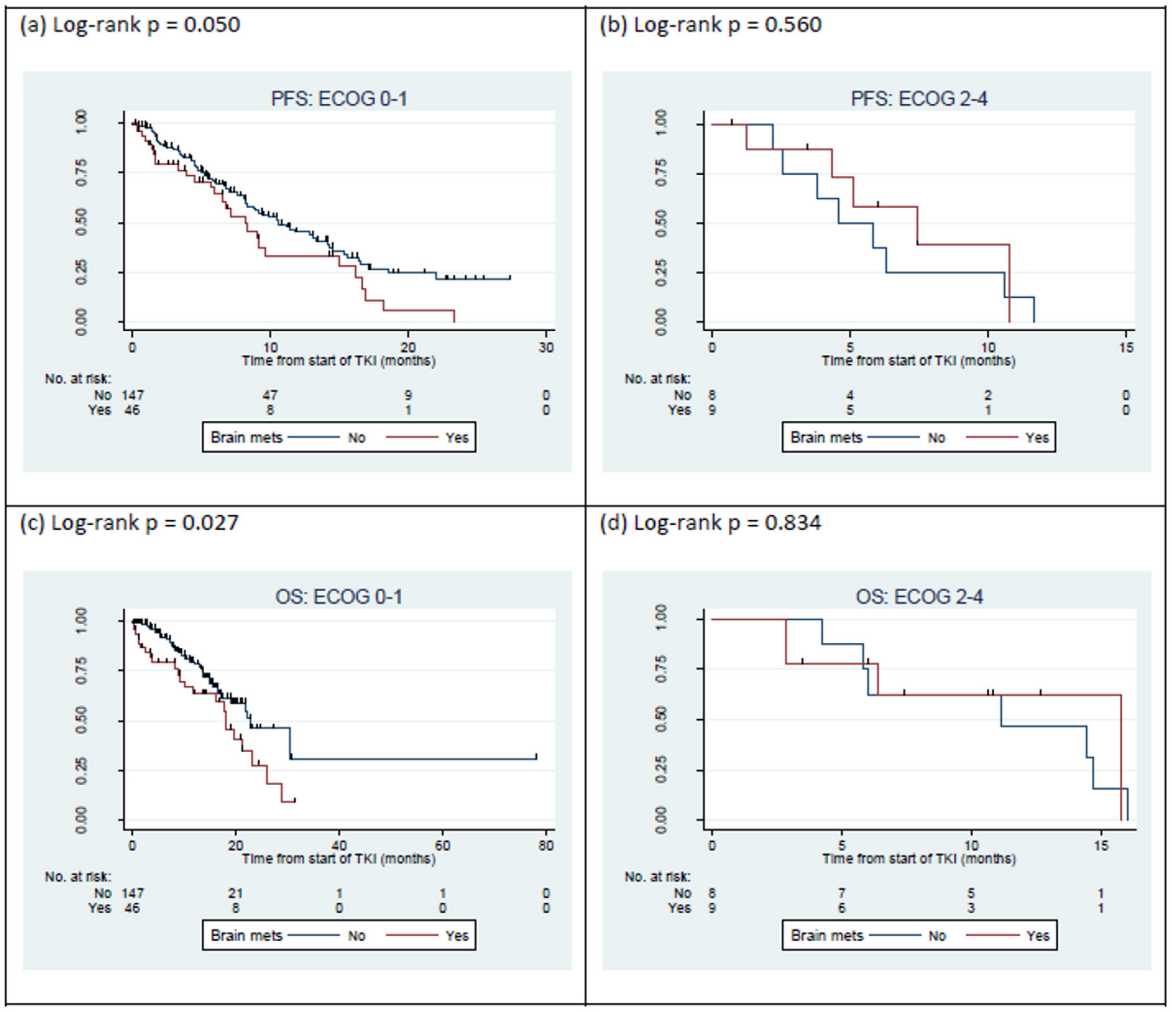
Kaplan-Meier plots of cohort of 211 patients treated with 1^st^ line EGFR TKI; (a) PFS by brain metastasis in ECOG 0–1 patients, (b) PFS by brain metastasis in ECOG 2–4 patients, (c) OS by brain metastasis in ECOG 0–1 patients, and (d) OS by brain metastasis in ECOG 2–4 patients.

On multivariate analysis, there was a trend for an inferior PFS in patients with brain metastasis at diagnosis (HR 1.56, CI 0.99–2.45, p = 0.053) (Table 3). Kaplan-Meier plots suggested that the presence of brain metastasis did not worsen progression free survival in patients who were ECOG 2–4 (Fig 4). This interaction between brain metastasis and ECOG status was incorporated into the multivariate model.

### Overall Survival of EGFR TKI treated Stage IV Disease

The overall median OS for all patients was 19.6 months (95% CI 16.4–23.3). The presence of brain metastasis at diagnosis was associated with a significantly worse OS in both univariate (p = 0.029), and multivariate (HR 1.82, CI 1.07–3.11, p = 0.028) analysis. Similar to findings on analysis for progression free survival, Kaplan-Meier plots suggested that the presence of brain metastasis did not worsen overall survival in patients who were ECOG 2–4, and this was incorporated into the multivariate model. The median OS of patients with no brain metastasis at diagnosis was 22.1 months while that of patients with brain metastasis at diagnosis was 17.9 months. Age, sex, ethnicity, smoking status (never vs. ever; never vs ex vs current; pack year < 50 vs pack year ≥ 50; pack year < 20 vs pack year ≥ 20), functional status, location and type of mutations had no significant impact on overall survival (Table 2). Multivariate analysis showed that smoking had no significant impact on overall survival (Table 3).

### Subset analyses in defined patient cohorts

Multivariate analysis of progression free survival and overall survival was performed amongst 2 defined patient cohorts to further investigate the effect of confounding factors. The first subset comprised 82 males (40 never smokers and 42 ever smokers) and was examined for the possible impact of smoking status on survival. Due to small sample size, the analysis was adjusted for ECOG status only. There was no evidence that smoking status was related to survival in this analysis (Table 4). A second subset comprised 70 female never smokers aged ≤ 65 with ECOG 0–1 at diagnosis (48 had no brain metastasis at diagnosis and 22 had brain metastasis at diagnosis) and was studied for the impact brain metastasis at diagnosis had on survival. After adjusting for type of mutation, brain metastasis at diagnosis had a significant impact on both progression free survival (HR 2.06, CI 1.03–4.11, p = 0.041) and overall survival (HR 2.86, CI 1.20–6.84, p = 0.018) (Table 5) in this cohort.

**Table 4:** Multivariate analysis of progression free survival and overall survival in males only.

Variable	No. of events / No. of patients	Hazard ratio (95% CI)	P-value	No. of events / No. of patients	Hazard ratio (95% CI)	P-value
	*Progression Free Survival*			*Overall Survival*		
Overall	42 / 82		0.166	31 / 82		0.073
Smoking status						
Never		1			1	
Ever		0.77 (0.41–1.43)	0.401		1.09 (0.54–2.22)	0.811
ECOG at diagnosis						
0–1		1			1	
2–4		2.31 (0.95–5.59)	0.064		3.05 (1.26–7.36)	0.013

**Table 5:** Multivariate analysis of progression free survival and overall survival in female never-smokers aged ≤ 65 with ECOG PS 0–1 at diagnosis.

Variable	No. of events / No. of patients	Hazard ratio (95% CI)	P-value	No. of events / No. of patients	Hazard ratio (95% CI)	P-value
	*Progression Free Survival*			*Overall Survival*		
Overall	37 / 70		0.087	24 / 70		0.006
Type of mutation						
Exon 19 deletion		1			1	
Exon 21 L858R mutation		0.98 (0.47–2.03)	0.958		0.28 (0.08–0.98)	0.047
Others		6.65 (0.74–59.45)	0.090		2.66 (0.73–9.69)	0.138
Brain metastasis at diagnosis						
No		1			1	
Yes		2.06 (1.03–4.11)	0.041		2.86 (1.20–6.84)	0.018

## Discussion

In our study, systematic testing of 762 lung adenocarcinoma for *EGFR* mutations revealed a prevalence of 61%—regardless of smoking status. Notably, we found that as high as 36% of male and 53% of female patients with a smoking history harboured *EGFR* mutations, and similar prevalence in males and females amongst never smokers (76.9% vs 75.1%)—underscoring the inadequacy of *EGFR* mutation testing based on clinical phenotype alone. This is especially relevant as cost-effectiveness of EGFR TKI is driven by patient selection based on mutation status, supporting reflex testing in our patient population regardless of smoking status and gender.

Our cohort of 211 *EGFR* mutant patients with stage IV NSCLC treated with first line EGFR TKI represents one of the largest to date examining prognostic factors. On multivariate analysis, taking into account *EGFR* mutations (exon 19 deletions versus L858R) and smoking status, the presence of brain metastasis at diagnosis—comprising 26% of our cohort—was an independent risk factor for worse overall survival, corroborating a recently reported retrospective series [16]. Although clinical activity of EGFR TKIs against intracranial disease has been previously described [17–20], most studies have comprised of modest-sized, heterogeneous and selected patient cohorts. Perhaps the most representative depiction of central nervous system (CNS) activity to date has been a retrospective series of 155 patients, where Heon et al. reported a lower 2-year cumulative risk of brain metastases in patients treated in the first line setting with an EGFR TKI (21%)—majority of whom (89%) received erlotinib—compared to chemotherapy (32%) [21]. In contrast, majority of patients in our study (91%) received gefitinib. It remains to be elucidated if erlotinib might exhibit superior control of intracranial disease due to higher CNS penetration and drug concentrations achieved compared to gefitinib [22]. Additionally, alternate treatment approaches such as pulsed high-dose strategies are actively being explored [23]. Further prospective studies addressing CNS disease are warranted, and should focus on improved delineation of leptomeningeal and cerebral metastases, quantity and quality of CNS disease (extent and symptoms), as well as optimal sequencing of EGFR TKI and radiation. Nevertheless, in the era of TKIs, *EGFR* M+ ADC patients with brain metastasis at diagnosis achieve a median survival of 17.9 months.

To date, there is limited data on the impact of smoking on progression free survival in patients treated with first line EGFR TKI. In three phase III trials that included up to 38% of ever smokers, subgroup analysis suggested no PFS benefit with TKI over conventional chemotherapy in contrast to those who had never smoked [5–7]. Retrospective studies evaluating the impact of smoking on the survival in *EGFR* M+ ADC [8, 24–26] have also yielded mixed results with only 1 study suggesting that smoking dosage has an impact on survival (Table 6). In our study of a homogenous cohort of patients with newly diagnosed metastatic *EGFR* mutation harbouring lung adenocarcinoma treated with EGFR TKI in the 1^st^ line, we found no evidence that smoking status or heavy smoking had a significant impact on PFS and OS, contrary to the recent report by Kim et al [8]. We acknowledge that our sample size was limited and that smoking status was confounded with gender, which we have tried to address. It is noteworthy that majority of smokers in the Korean study received EGFR TKI in the setting of second, third line or beyond, where cumulative toxicities and poor functional status may confound outcomes. The presence or absence of brain metastasis was also not reported in that study.

**Table 6:** Hazards for survival from univariate analysis of populations with smoking characteristics as indicated across 3 studies do not show any significant differences in survival outcomes, while those from a more recent Korean study show that smoking has a significant impact, especially smoking dosage greater than 30 pack years.

Study	Smoking Characteristics	Progression Free Survival			Overall Survival		
		HR	95% CI	P value	HR	95% CI	P value
Rosell et al. [24]	Former vs. Never	0.72	0.39–1.32	0.29	0.70	0.32–1.54	0.38
	Current vs. Never	1.65	0.69–3.96	0.26	1.37	0.45–4.22	0.58
Paik et al. [23]	Smoker vs. Never	-	-	-	-	-	0.33
Morita et al. [25]	Smoker vs. Never	1.08	0.60–1.96	0.794	1.22	0.62–2.38	0.570
Kim et al. [8]	Ever vs. Never	1.47	1.07–2.03	0.018	1.73	1.20–2.48	0.003
	<30 pack years vs. Never	1.15	0.77–1.74	0.497	1.51	0.95–2.40	0.800
	> 30 pack years vs. Never	2.03	1.35–3.06	0.001	2.00	1.27–3.16	0.003

The retrospective nature of our study limits our ability to evaluate on-treatment CNS activity for EGFR TKI, due to irregular frequency and thoroughness of follow-up radiological evaluation. In addition, there may yet be unaccounted bias between those with and without brain metastases, although the commonly reported factors such as performance status, smoking status and type of *EGFR* mutation were addressed. To our knowledge, this is the first study examining these clinical parameters in a large *EGFR* M+ patient cohort predominantly treated with first line gefitinib.

In conclusion, our study highlights the importance of reflex molecular testing, where as high as 36% of ever smoker males were found to harbour an *EGFR* mutation. Furthermore, despite the higher mutational burden in smokers [27], the observation that smoking status did not impact on PFS or OS in patients treated with first line EGFR TKI, suggests a hierarchical relationship in genetic alterations, where dominant truncal events—such as *EGFR* mutations—remain therapeutically tractable. On the other hand, the presence of brain metastases emerged as an independent negative prognostic factor, and continues to be a therapeutic challenge in *EGFR* M+ NSCLC.

## Supporting Information

S1 Table Reflex testing of all newly lung adenocarcinoma cases for mutations in *EGFR* was commenced from 1^st^ June 2010, with current rates of testing between 87 to 94%. *EGFR* mutations were found in 50–68% of cases. Prior to the implementation of reflex testing, patients were tested for *EGFR* mutations based on physician discretion. A total of 742 patients underwent *EGFR* mutation testing. 444 (59.8%) were positive and 289 (38.9%) negative for mutations. 9 cases were unsuccessfully profiled. Hence the ascertainment rate with *EGFR* sequencing in our centre was 98.8%.
(DOCX)

S2 Table Baseline characteristics of Never Smokers vs. Ever Smokers amongst 211 patients treated with 1^st^ line TKI. (DOCX)

S3 Table Baseline characteristics of patients without brain metastasis at diagnosis vs. those with brain metastasis at diagnosis amongst 211 patients treated with 1^st^ line TKI. (DOCX)
