# Supplementary material for: Impact of Smoking and Brain Metastasis on Outcomes of Advanced EGFR Mutation Lung Adenocarcinoma Patients Treated with First Line Epidermal Growth Factor Receptor Tyrosine Kinase Inhibitors
Source: PLoS One. 2015 May 8;10(5):e0123587. doi: 10.1371/journal.pone.0123587 (PMC4425557; doi:10.1371/journal.pone.0123587)
Supplement: S1 Table — EGFR mutations were found in 50–68% of cases. Prior to the implementation of reflex testing, patients were tested for EGFR mutations based on physician discretion. A total of 742 patients underwent EGFR mutation testing. 444 (59.8%) were positive and 289 (38.9%) negative for mutations. 9 cases were unsuccessfully profiled. Hence the ascertainment rate with EGFR sequencing in our centre was 98.8%. (DOCX) [file pone.0123587.s001.docx]

**Table S1** – Reflex testing of all newly lung adenocarcinoma cases for mutations in *EGFR* was commenced from 1^st^ June 2010, with current rates of testing between 87 to 94%. *EGFR* mutations were found in 50-68% of cases. Prior to the implementation of reflex testing, patients were tested for *EGFR* mutations based on physician discretion. A total of 742 patients underwent *EGFR* mutation testing. 444 (59.8%) were positive and 289 (38.9%) negative for mutations. 9 cases were unsuccessfully profiled. Hence the ascertainment rate with *EGFR* sequencing in our centre was 98.8%.

(ADC = Adenocarcinoma)

| **Period** | **No. (%) Lung ADC tested for *EGFR* Mutations** | **No. (%) *EGFR* M +** |
| --- | --- | --- |
| 1^st^ January to 31^st^ May 2009 | 8/70 (11%) | 3/8 (38%) |
| 1^st^ June to 31^st^ December 2009 | 36/91 (39%) | 26/36 (72%) |
| 1^st^ January to 31^st^ May 2010 | 50/91 (54%) | 28/50 (56%) |
| 1^st^ June to 31^st^ December 2010 | 155/174 (89%) | 77/155 (50%) |
| 1^st^ January to 31^st^ May 2011 | 114/123 (93%) | 77/114 (68%) |
| 1^st^ June to 31^st^ December 2011 | 137/146 (94%) | 92/137 (67%) |
| 1^st^ January to 31^st^ May 2012 | 89/100 (89%) | 53/89 (60%) |
| 1^st^ June to31^st^ December 2012 | 107/123 (87%) | 63/107 (59%) |
| 1^st^ January to19^th^ April 2013 | 46/51 (90%) | 25/46 (54%) |
