# Supplementary material for: Impact of Smoking and Brain Metastasis on Outcomes of Advanced EGFR Mutation Lung Adenocarcinoma Patients Treated with First Line Epidermal Growth Factor Receptor Tyrosine Kinase Inhibitors
Source: PLoS One. 2015 May 8;10(5):e0123587. doi: 10.1371/journal.pone.0123587 (PMC4425557; doi:10.1371/journal.pone.0123587)
Supplement: S2 Table — (DOCX) [file pone.0123587.s002.docx]

**Table S2 –** Baseline characteristics of Never Smokers vs. Ever Smokers amongst 211 patients treated with 1^st^ line TKI.

| **Variable** | **Never Smoked (%)** | **Ever Smoked (%)** | **Correlation** | **P-value** |
| --- | --- | --- | --- | --- |
| Overall | 166 (78.7) | 45 (21.3) |  |  |
| Age |  |  |  |  |
| ≤ 65 | 108 (65.1) | 20 (44.4) | 0.173 | 0.012 |
| > 65 | 58 (34.9) | 25 (55.6) |  |  |
|  |  |  |  |  |
| Gender |  |  |  |  |
| Female | 125 (75.3) | 3 (6.7) | 0.576 | < 0.001 |
| Male | 41 (24.7) | 42 (93.3) |  |  |
|  |  |  |  |  |
| Brain metastasis at diagnosis  diagnosis |  |  |  |  |
| No | 124 (74.7) | 32 (71.1) | 0.034 | 0.627 |
| Yes | 42 (25.3) | 13 (28.9) |  |  |
| ECOG at diagnosis |  |  |  |  |
| 0 – 1 | 155 (93.4) | 39 (86.7) | 0.101 | 0.211 |
| 2 – 4 | 11 (6.6) | 6 (13.3) |  |  |
|  |  |  |  |  |
| Type of mutation |  |  |  |  |
| Exon 19 deletion | 96 (57.8) | 18 (40.0) | 0.173 | 0.044 |
| Exon 21 L858R mutation | 54 (32.5) | 18 (40.0) |  |  |
| Others | 13 (7.8) | 8 (17.8) |  |  |
| Unknown | 3 (1.8) | 1 (2.2) |  |  |
