# Supplementary material for: Impact of Smoking and Brain Metastasis on Outcomes of Advanced EGFR Mutation Lung Adenocarcinoma Patients Treated with First Line Epidermal Growth Factor Receptor Tyrosine Kinase Inhibitors
Source: PLoS One. 2015 May 8;10(5):e0123587. doi: 10.1371/journal.pone.0123587 (PMC4425557; doi:10.1371/journal.pone.0123587)
Supplement: S3 Table — (DOCX) [file pone.0123587.s003.docx]

**Table S3 –** Baseline characteristics of patients without brain metastasis at diagnosis vs. those with brain metastasis at diagnosis amongst 211 patients treated with 1^st^ line TKI.

| **Variable** | **No Brain Mets** | **Brain Mets** | **Correlation** | **P-value** |
| --- | --- | --- | --- | --- |
| Overall | 156 (73.9) | 55 (26.1) |  |  |
| Age |  |  |  |  |
| ≤ 65 | 87 (55.8) | 41 (74.5) | 0.169 | 0.014 |
| > 65 | 69 (44.2) | 14 (25.5) |  |  |
|  |  |  |  |  |
| Gender |  |  |  |  |
| Female | 94 (60.3) | 34 (61.8) | 0.014 | 0.838 |
| Male | 62 (39.7) | 21 (38.2) |  |  |
|  |  |  |  |  |
| Smoking status |  |  |  |  |
| Never | 124 (79.5) | 42 (76.4) | 0.034 | 0.627 |
| Ever | 32 (20.5) | 13 (23.6) |  |  |
|  |  |  |  |  |
| ECOG at diagnosis |  |  |  |  |
| 0 – 1 | 148 (94.9) | 46 (83.6) | 0.181 | 0.017 |
| 2 – 4 | 8 (5.1) | 9 (16.4) |  |  |
|  |  |  |  |  |
| Type of mutation |  |  |  |  |
| Exon 19 deletion | 81 (51.9) | 33 (60.0) | 0.073 | 0.578 |
| Exon 21 L858R mutation | 56 (35.9) | 16 (29.1) |  |  |
| Others | 16 (10.3) | 5 (9.1) |  |  |
| Unknown | 3 (1.9) | 1 (1.8) |  |  |
